# Supplementary material for: Viral load suppression and its predictor among HIV seropositive people who receive enhanced adherence counseling at public health institutions in Bahir Dar, Northwest Ethiopia. Retrospective follow-up study
Source: PLoS One. 2024 May 13;19(5):e0303243. doi: 10.1371/journal.pone.0303243 (PMC11090359; doi:10.1371/journal.pone.0303243)
Supplement: S1 Appendix — (PDF) [file pone.0303243.s001.pdf]

Appendix 1. Information Sheet

Title of the Research: - Viral load suppression and its predictor after enhanced adherence counseling among HIV seropositive people with high viral load at Bahir Dar city public health institution, Northwest Ethiopia 2021.

Name of Investigator: Minyichil Birhanu Belete

Name of the Organization: Bahir Dar University College of Medicine and Health Sciences, School of Public Health, Department of Epidemiology and Biostatistics

Introduction: This information sheet was prepared for each public Health institution administration staff and ART focal person. This information aimed to make the above-concerned office clear about the purpose of research, data collection procedures, and get permission to carry out the research.

Purpose of the Research: To assess viral load suppression and its predictor after enhanced adherence counseling among HIV seropositive people with high viral load at Bahir Dar city public health institution, Northwest Ethiopia 2021.

Procedure: To achieve the above objective, information that was necessary for the study will be taken from the patient's medical record chart, enhanced adherence counseling sheet, viral load registration book, and laboratory request.

Risk and or Discomfort: Since the study was done by taking appropriate information from medical records, it did not impose any harm on the patients. The name of patients or any other personal identifying information was not recorded on the extraction checklist. All of the information which was taken from the patient's chart was kept in a safe place and strictly confidential. The retrieved information will be used only for the study purpose.

Benefits: This research has no direct benefit for one whose medical record was included in the research. However, the indirect benefit of the research for the study participants and other patients in the program was clear. This is due to that if the program planners are preparing a predicted plan there is a benefit for patients with high viral load to gate appropriate care and treatment services. Hence findings from this study will add evidence-based information especially to the study area where limited study as to my knowledge and have implications on designing and implementing health policies and programs.

Confidentiality: -To reassure confidentiality, data was collected without the name of the patients, and the information collected for this research project was kept confidential and stored in a file

cabinet. The information obtained from the chart was stored in a file with a code number assigned to it and it was not revealed to anyone except the principal investigator and it was kept in a key and locked system with a computer pass ward.

Person to contact: This research project was reviewed and approved by the institutional review board of the College of Medicine and Health Sciences, Bahir Dar University. Anyone who has an issue can contact any of the Investigator and/or Advisors at any time using the address below.

PI: Minyichil Birhanu Belete

Cell phone: +251 911 58 65 25

Email: mbym24@gmail.com

Advisors:

1. Mr. Abebayehu Bitew (MPH, Assistant Professor)

   Email: abebayehubitew@gmail.com

2. Mr. Kebadnew Mulatu (MPH, Assistant Professor)

   Email: kebadmulat@gmail.com
