# Supplementary material for: Viral load suppression and its predictor among HIV seropositive people who receive enhanced adherence counseling at public health institutions in Bahir Dar, Northwest Ethiopia. Retrospective follow-up study
Source: PLoS One. 2024 May 13;19(5):e0303243. doi: 10.1371/journal.pone.0303243 (PMC11090359; doi:10.1371/journal.pone.0303243)
Supplement: S2 Appendix — (PDF) [file pone.0303243.s002.pdf]

Appendix 2. Data Collection Tools

This data extraction tool was prepared to collect socio-demographic, Baseline clinical and laboratory-related, treatment-related factors, and other related information that was important for the assessment of viral load suppression and its predictors among HIV seropositive clients with high viral load and enrolled in EAC from January 2017 to December 2021 at public health facilities in Bahir Dar city, Northwest Ethiopia. All this information will be retrieved from the patient's medical record chart, enhanced adherence counseling sheet, viral load registration book, and laboratory request without mentioning the name. The data will be collected by health care providers who had a BSC degree in Nursing.

Part I. Socio-demographic Characteristics

S.No	Questions	Possible Answers	Remark/ Skip to
101.	What was the Age of the client at the time of High viral load detected?	__________ years	
102.	What is the sex of the client?	1. Male<br>2. Female	
103.	Where is the residency of the client?	1. Urban<br>2. Rural	
104.	What is his/her religion?	1. Orthodox Tewahido<br>2. Muslim<br>3. Protestant<br>4. Catholic<br>5. Others specify________	
105.	In which type of health facility does the client receive enhanced adherence counseling?	1. Specialized hospital<br>2. Primary hospital<br>3. Health center	
106.	What is the marital status of the client?	1. Never married<br>2. Married<br>3. Divorced<br>4. Widowed	If the answer is 1,3,4 skip to 108
107.	What is a client's spouse HIV status?	1. Positive<br>2. Negative<br>3. Currently single	

		4. Not applicable	
108.	Occupation	1. Government employee<br>2. Farmer<br>3. Daily laborer<br>4. Merchant<br>5. Private work<br>6. House wife<br>7. Others specify________	
109.	Educational status	1. Cannot read and write<br>2. Able to read and write only<br>3. Primary<br>4. Secondary<br>5. College and above<br>6. Others specify________	
110.	Baseline body weight	__________ kg	
111.	Baseline Height	__________ cm	
112.	Baseline Body mass index	_________(kg/m^2)	

Part II: Baseline Clinical and Laboratory Related Characteristics

S.No.	Questions	Possible Answers		Remark/Skip to
201.	Baseline CD4 count	____________cell/mm^3		
202.	Baseline viral load count at enrollment to EAC (1st VL result)	________ copies/ml	Date:__/___/____	
203.	Baseline WHO clinical stage	1. Clinical stage I<br>2. Clinical stage II<br>3. Clinical stage III<br>4. Clinical stage IV		
204.	Functional status	1. Working<br>2. Ambulatory<br>3. Bedridden		
205.	Presence of recurrent opportunistic infections before high viral load detected	0. No<br>1. Yes		If "No" skip to "207"
206.	Opportunistic infection present	1. Oral Candidiasis		

		2. Tuberculosis<br>3. Herpes Zooster<br>4. Pneumonia<br>5. Others specify__________		
207.	Do you have a hospital admission history?	0. No<br>1. Yes		
208.	Date of first EAC session	____/____/___________		
209.	Date of last EAC session	____/____/___________		
210.	Time gap between high viral load detected to the EAC session started?	_______________ days		
211.	Total number of EAC sessions	__________		
212.	Number of dates taken to complete EAC sessions	_______________ days		
213.	Time gap between last EAC session and 2nd viral load done(received)	_______________ days		
214.	2nd Viral Load result	________ copies/ml	Date:__/___/____	
215.	3rd Viral Load result	________ copies/ml	Date:__/___/____	
216.	Twelve Months VL Result	________ copies/ml	Date:__/___/____	
217.	What was the viral load count at the end of EAC?	___________copies/mm^3		
218.	What was the viral load status at the end of EAC?	1. Suppressed (≤1000 copies/ml)<br>2. Unsuppressed(>1000 copies/ml)		

Part III: Behavioral and social related Characteristics

S no	Questions	Possible answers	Remark/ skip to
301.	Have you ever disclosed your HIV status to your family?	0. No<br>1. Yes	
302.	Have your families been non-supportive/kept you from taking ARVs?	0. No<br>1. Yes	
303.	Are you confident to take your ARVs openly at home?	0. No<br>1. Yes	

304.	**Did you correctly and consistently use condom during sexual intercourse?**	0. No<br>1. Yes<br>2. Not applicable	
305.	In the past 3 months; Was there ever a time when you felt sad or hopelessness for more than 2 weeks in a row?	0. No<br>1. Yes	
306.	In the past 3 months; Was there ever a time lasting more than 2 weeks when you lost interest in most things like hobbies, work, or activities that usually give you pleasure?	0. No<br>1. Yes	
307.	Do you take alcohol or Khat?	0. No<br>1. Yes	
308.	Has a lack of food ever been a problem for taking your ARVs?	0. No<br>1. Yes	
309.	Have you discontinued your ARVs to take other remedies for HIV?	0. No<br>1. Yes	
310.	Did the client attend all EAC sessions regularly?<br>If no, any reason?	0. No<br>1. Yes	If "1" skip to "401"
311.	The reason for not attend all EAC sessions regularly	______________	

Part IV: Treatment related characteristics

S.No	Questions	Possible answers	Remark/skip to
401.	Currently, at which ART regimen the client does?	1. 1st line<br>2. 2nd line<br>3. 3rd line	
402.	In which specific ART regimen does the client is?	1. TDF/3TC/EFV<br>2. AZT/3TC/NVP<br>3. ABC/3TC/EFV<br>4. TDF/3TC/ATVr<br>5. TDF/3TC/LPVr<br>6. AZT/3TC/ATVr<br>7. ABC/3TC/ATVr<br>8. ABC/3TC/LPVr<br>9. Others specify______	
403.	For how long the client is on ART?	_______ months	

404.	Does the client take INH?	0. No<br>1. Yes	
405.	Does the client take CPT	0. No<br>1. Yes	
406.	How many ARV doses do you take/day in the last 1month?	1. Once<br>2. Twice	
407.	Did you miss ARV doses in the past one month?	0. No<br>1. Yes	If "0" skip to "410"
408.	If Yes, the number of doses missed in the past month? (if the client takes once daily regimen)	1. <2 doses<br>2. 2-4 doses<br>3. ≥5 doses	
409.	If yes, the number of doses missed in the past month? (if the client takes twice daily regimen)	1. ≤ 3 doses<br>2. 4-9 doses<br>3. >9 doses	
410.	Adherence measurement during EAC	1. Good<br>2. Fair<br>3. Poor	
411.	Have you ever missed ARV doses in the past since you started ART?	0. No<br>1. Yes	
412.	**If ARV doses missed in the past since you started ART,** What was the reason?	______________	
413.	Have you ever discontinued/interrupted care follow up/medication refill since you started ART?	0. No<br>1. Yes	
414.	If you discontinued/interrupted care follow up /medication refill since you started ART, What was the reason?	______________	
415.	Did you forget taking ARVs doses at a scheduled time/day?	0. No<br>1. Yes	
416.	Did you forget to give your child the prescribed dose/s of ARVs at a scheduled time/day?	0. No<br>1. Yes	
417.	Did you Directly Observe your child when he/she taking ARVs doses at a scheduled time/day?	0. No<br>1. Yes	
