# Supplementary material for: Viral load suppression and its predictor among HIV seropositive people who receive enhanced adherence counseling at public health institutions in Bahir Dar, Northwest Ethiopia. Retrospective follow-up study
Source: PLoS One. 2024 May 13;19(5):e0303243. doi: 10.1371/journal.pone.0303243 (PMC11090359; doi:10.1371/journal.pone.0303243)
Supplement: S3 Appendix — (PDF) [file pone.0303243.s003.pdf]

Appendix 3. Declaration form

DECLARATION

I Minyichil Birhanu, hereby declare that to the best of my information this research thesis is my work; it has not been presented to any institution either partially or in total for any purposes. The works herein are original, where the works of others are acknowledged through appropriate referencing.

This research thesis is needed for partial fulfillment of the Degree of Master of Public Health in Epidemiology.

Principal Investigator:

Mr. Minyichil Birhanu **Signature**: ____________ **Date:** August 17, 2022

This research thesis will be submitted with the approval of advisors and examiners:

Advisors:

1. **Mr. Abebayehu Bitew** (MPH, Assistant Professor) **Signature**: ______ **Date**: August 17, 2022

2. **Mr. Kebadnew Mulatu** (MPH, Assistant Professor) **Signature**: ______ **Date**: August 17, 2022
