# Supplementary material for: Viral load suppression and its predictor among HIV seropositive people who receive enhanced adherence counseling at public health institutions in Bahir Dar, Northwest Ethiopia. Retrospective follow-up study
Source: PLoS One. 2024 May 13;19(5):e0303243. doi: 10.1371/journal.pone.0303243 (PMC11090359; doi:10.1371/journal.pone.0303243)
Supplement: S4 Appendix — (PDF) [file pone.0303243.s004.pdf]

Appendix 4. Examiner's approval form

EXAMINER'S APPROVAL FORM

BAHIR DAR UNIVERSITY

COLLEGE OF MEDICINE AND HEALTH SCIENCES

SCHOOL OF PUBLIC HEALTH

DEPARTMENT OF EPIDEMIOLOGY AND BIOSTATISTICS

Approval of Thesis Report

I hereby certify that I have examined this thesis report entitled "Viral Load Suppression and Its Predictor among HIV Seropositive People Who Receive Enhanced Adherence Counseling at Public Health Institutions in Bahir Dar, Northwest Ethiopia. Retrospective Follow-up Study." by Mr. Minyichil Birhanu. We recommend and approve the thesis report for a "DEGREE OF MASTER OF PUBLIC HEALTH IN EPIDEMIOLOGY".

Board of Examiners

______________________	__________	**August 17, 2022**
External examiner's name	Signature	Date
**Mr. Zelalem Mehari** (MSc, Assistant Professor)	[signature]	**August 17, 2022**
Internal examiner's name	Signature	Date
**Mr. Zelalem Alamrew** (MPH, Assistant Professor)	[signature]	**August 17, 2022**
Chair person's name	Signature	Date

Department of Epidemiology and Biostatistics
