# Supplementary material for: Viral load suppression and its predictor among HIV seropositive people who receive enhanced adherence counseling at public health institutions in Bahir Dar, Northwest Ethiopia. Retrospective follow-up study
Source: PLoS One. 2024 May 13;19(5):e0303243. doi: 10.1371/journal.pone.0303243 (PMC11090359; doi:10.1371/journal.pone.0303243)
Supplement: Attachment — Submitted filename: Viral load suppression.pdf [file pone.0303243.s005.pdf]

Comments

Thank you for the opportunity to review the manuscript entitled "viral load suppression and its predictors among HIV-seropositive who received Enhanced adherence counseling at public health institution in Bahir Dar, Northwest Ethiopia. Retrospective follow-up study.

I think this study provides information predictors among HIV-seropositive who received Enhanced adherence counseling at public health institution, and this study might be an excellent input for the national government for decision making.

Please see the comments and respond accordingly below before considering this manuscript suitable for publication.

1. I do have an issue on your title; many studies have carried out related to these topics what makes add for the scientific communities.
2. From the abstract section the **background** section is too long you should have state your research question or your problem 2 to 3 statements
3. In your conclusion part you have described that "the majority of individuals had decreased viral loads" what is your parameters to say decreased or have done any comparison group
4. Generally your abstract section too long, make it short as much as you possible.
5. From your introduction section you have wrote greater than seven pages, since it's a scientific paper you should have narrate to one page unless maximum two page by focusing your research question and significance your study, unless its seems a negligence of the authorship.
6. On your document on page 4, 6 &24 your stating "In September 2020, UNAIDS reported a gap in achieving its 90-90-90 global targets", its an old evidence wrote here please the current evidence on UNAIDS global target plan.
7. I doubt a clarity question about on your methodology section especially your study design (really retrospective follow-up study or not??), if its right I have a lot question rises on your variables, where you do get behavioral related question and others?
8. Your study, target population is does not clearly explained (adult, adolescence, or pediatrics age group)
9. Could you think its enough single population proportion formula for sample size determination, if not why you are not stated here?

10. I think it’s better to write or define enhanced adherence counseling(EAC) on your operational definition
11. Again Behavioral related predictors(Cigarette smoking, alcohol drinking, khat chewing) were present in the intake form as well in the follow-up form, even also its difficult addressed from your Treatment related factors such as time gap from high viral load detected to EAC session start, time to complete EAC sessions, history of drug discontinuation. **Its A big Issue for me needs intense response unless it might be return back re-analysis.**
12. If your data collection were in electronic based platform what its need of supervisor, how do you managed information contamination since you have shared your data for your data collectors and supervisors?
13. Since you have generated or collect five years retrospective data how much really your variables is feasible to avail such variables **Source of referral variables??Depressed???**

Am happy the way you state result section

14. Minor comment on page 17 sub topics **“Proportion of unsuppressed Viral Load”** makes it suppressed unless the body of the statement seems paradox, or make it correct the statement.
15. One of your predictors for viral load suppression is “being Orthodox Tewahido Christian” which is “decreases the probability of achieving viral load suppression after EAC”, so what do you recommend to scientific community, international community. In my point of view this variables is not necessary to state here as predictors. Unless its strong evidence to recommendation.
16. Your conclusion “ this study reported that viral load suppression after EAC was found high”, what is your evidence to say “HIGH”
