# Supplementary material for: Viral load suppression and its predictor among HIV seropositive people who receive enhanced adherence counseling at public health institutions in Bahir Dar, Northwest Ethiopia. Retrospective follow-up study
Source: PLoS One. 2024 May 13;19(5):e0303243. doi: 10.1371/journal.pone.0303243 (PMC11090359; doi:10.1371/journal.pone.0303243)
Supplement: Attachment — Submitted filename: Response to Reviewers.docx [file pone.0303243.s006.docx]

**Response to Reviewers**

**Reviewer #1**

I do have an issue on your title; many studies have carried out related to these topics what makes add for the scientific communities.

**Response:**

There are a few studies in the area of viral load suppression after EAC. However, there is not a single study in our study area. Additionally, some of the papers are focused on the adult age group only. Our study area was a specialized referral hospital that could include a population on the 3rd ART regimen, whereas the previous studies focused on HIV-infected people on the first-line regimen only.

**Reviewer #1**

From the abstract section the background section is too long you should have state your research question or your problem 2 to 3 statements

**Response:**

We try to summarize the background session of the abstract

**Reviewer #1**

In your conclusion part you have described that “the majority of individuals had decreased viral loads” what is your parameters to say decreased or have done any comparison group

**Response:**

We say the majority based on the finding that we go which was 312 (57.1%) which is more than the half of the participants

**Reviewer #1**

From your introduction section you have wrote greater than seven pages, since it’s a scientific paper you should have narrate to one page unless maximum two page by focusing your research question and significance your study, unless it seems a negligence of the authorship.

**Response**

Thank you for your feedback on my paper. I appreciate your suggestion regarding the length of the introduction section. I understand that scientific papers typically have shorter introduction sections and I apologize for any inconvenience my longer introduction may have caused you.

I assure you that I have not neglected my responsibilities as an author and have taken great care to ensure that my paper is of high quality and meets the standards of the journal and I resynthesis the introduction part. I appreciate your time and effort in reviewing my paper and welcome any further feedback or suggestions you may have.

**Reviewer #1**

I doubt a clarity question about on your methodology section especially your study design (really retrospective follow-up study or not??), if its right I have a lot question rises on your variables, where you do get behavioral related question and others?

**Response:**

My study design was a retrospective follow-up study, and the standardized adherence counseling format incorporates variables regarding behavioral-related questions such HIV status disclosure status, quitting ARV to take alternative treatments, Having a non-supportive family, being confident to take ARV openly at home, having a current sexual status, condom utilization status, depression status, substance use, and a lack of food are obstacles with taking ARV, etc. associated difficulties are clearly indicated in the adherence counseling, which will be filled out in all appointments.

**Reviewer #1**

Your study, target population is does not clearly explained (adult, adolescence, or pediatrics age group)

**Response**

The target population of our study was all age group HIV seropositive people received enhanced adherence counseling at public health facilities

**Reviewer #1**

Could you think it’s enough single population proportion formula for sample size determination, if not why you are not stated here?

**Response**

At the design stage of our study, we compute our sample size using both the double population proportion formula and the single population proportion formula, but we get the greatest sample size when we utilize the single population proportion calculation. And that is why we put only the formula that we last utilized.

**Reviewer #1**

I think it’s better to write or define enhanced adherence counseling (EAC) on your operational definition

**Response**

We operationalize what is Enhanced Adherence Counseling

**Reviewer #1**

Again, Behavioral related predictors (Cigarette smoking, alcohol drinking, khat chewing) were present in the intake form as well in the follow-up form, even also its difficult addressed from your Treatment related factors such as time gap from high viral load detected to EAC session start, time to complete EAC sessions, history of drug discontinuation. Its A big Issue for me needs intense response unless it might be return back re-analysis.

**Response**

We collect our data from the high viral load registration booklet, intake form, EAC follow-up form, and client medical card. With this all-data source, we assure that we can access all the data related to behavioral-related predictors (cigarette smoking, alcohol drinking, and khat chewing), treatment-related factors such as the time gap from high viral load detected to EAC session start, time to complete EAC sessions, and history of drug discontinuation. All this data is readily available from the above-mentioned data sources that we employ.

**Reviewer #1**

If your data collection were in electronic based platform what its need of supervisor, how do you managed information contamination since you have shared your data for your data collectors and supervisors?

**Response**

We collect data using an electronic platform, and supervisors were recruited to observe the quality of data at each data collection site and to examine the daily data collected by each data collector before the data is uploaded to the server.

Regarding the information contamination, information contamination is not a concern for us since we are using secondary data.

**Reviewer #1**

Since you have generated or collect five years retrospective data how much really your variables is feasible to avail such variables Source of referral variables??Depressed???

**Response**

As the authors of this study, we ensure that all the data characteristics were assured as much as feasible. Additionally, Enhanced Adherence Counseling for HIV seropositive patients with a high viral load is a well-structured program that is actively supported, and data quality is monitored and assessed by CDC professionals recruited there. With all these guidelines, we can assure that all the data we utilize is clearly documented in the data source that we use.

**Reviewer #1**

One of your predictors for viral load suppression is “being Orthodox Tewahido Christian” which is “decreases the probability of achieving viral load suppression after EAC”, so what do you recommend to scientific community, international community. In my point of view these variables is not necessary to state here as predictors. Unless its strong evidence to recommendation.

**Response**

Based on our findings, the majority of the study participants were Orthodox Tewahido Christians, and they believe Holy Water gives a cure for HIV. Consequently, they discontinue their ARV medication and follow up for Holy Water service. As evidenced by the EAC follow-up form, the majority of those clients who discontinued ART were to go to Holy Water Service. So, our recommendation is to counsel them so they can take ART in parallel with holy water instead of discontinuing the drug. And we put our recommendation in the recommendation session on lines 488 to 489 as “And also, it is better to strengthen the engagement of Orthodox Tewahido religious leaders in ART care programs.”

**Reviewer #1**

Your conclusion “this study reported that viral load suppression after EAC was found high”, what is your evidence to say “HIGH”

**Response**

We see your concern, and it is a typographic error. We consider your feedback and correct it accordingly.

**Reviewer #2:**

Title: your title is not novel, because there are recent similar studies conducted in north Wollo and west Gojam.

**Response**

We cited both research findings in our study; however, the study in the north Wollo zone focused primarily on HIV-infected patients on the first-line regimen. Whereas the study done in west Gojam focused mainly on HIV-positive adult patients on ART. Thus, the current study covers clients in all regimens and all age categories.

**Reviewer #2:**

Abstract: the number of words in this section is beyond the scientifically acceptable limit and it should be summarized in one page.

**Response**

We welcome your suggestion, and we summarize the abstract part accordingly.

**Reviewer #2:**

Introduction: it is vague (more than 7 pages) and seeming literature review, please try to summarize with no more than 4 pages in concise manner.

**Response**

We accept your valuable feedback and we amend accordingly

**Reviewer #2:**

Methods and materials: in your study area part better to describe how many HIV sero-positive people are served and reduce other unnecessary statements especially those stated in line no 220-222. Are your study, source and target population similar? If yes, write them separately.

**Response**

In our research, the target population and study population were not similar, and we described them separately according to your recommendation.

**Reviewer #2:**

Result and Discussion: Good, but I doubt whether all the significant variables of the study are discussed properly since they are excess, i.e., six.

**Response**

Thank you for your feedback on the results and discussion section of our study. We appreciate your comment on the number of significant variables, and we would like to clarify that all variables were discussed in detail, although we understand that there may be an excess.

In our study, we aimed to investigate the relationship between several variables and the outcome of interest. Through our analysis, we found five variables to be significant predictors of the outcome. In our discussion, we provided a comprehensive overview of each significant variable, including its theoretical relevance, effect size, and direction of association with the outcome. We also discussed the potential mechanisms underlying these associations and their implications for future research and practice.

However, we understand that the discussion of multiple variables can be complex and overwhelming. To address this issue, we have revised our discussion section to provide a more concise and focused overview of each variable, highlighting their unique contributions to the outcome. We hope that this revised version better addresses your concerns and provides a clearer understanding of our findings. Thank you again for your valuable feedback.

**Reviewer #2:**

In the result section make some of the tables in figure form to make the study more attractive.
Strength and Limitation: the same idea is written on line number 454 and 458. Better to consider it as limitation of your study.

**Response**

When evaluating our research, we identified the data sources as a key strength and highlighted them as the primary level of documentation. However, despite their importance, we acknowledged that they are not without flaws, which we discussed in the limitations section. It's worth noting that the concepts we covered in the strength and limitation parts are distinct from one another.

**Reviewer #2:**

Conclusion and recommendation: conclude your outcome variable, like; suppressed or not suppressed, low or high. The recommendation should be summarized with no more than three sentences.

**Response**

We accept your feedback, and we amend accordingly.
