# Supplementary material for: Viral load suppression and its predictor among HIV seropositive people who receive enhanced adherence counseling at public health institutions in Bahir Dar, Northwest Ethiopia. Retrospective follow-up study
Source: PLoS One. 2024 May 13;19(5):e0303243. doi: 10.1371/journal.pone.0303243 (PMC11090359; doi:10.1371/journal.pone.0303243)
Supplement: Attachment — Submitted filename: Response to Reviewers.docx [file pone.0303243.s007.docx]

**Response to Reviewers**

**Journal editor:**

Please review your reference list to ensure that it is complete and correct. If you have cited papers that have been retracted, please include the rationale for doing so in the manuscript text, or remove these references and replace them with relevant current references. Any changes to the reference list should be mentioned in the rebuttal letter that accompanies your revised manuscript. If you need to cite a retracted article, indicate the article’s retracted status in the References list and also include a citation and full reference for the retraction notice.

**Response:**

We made amendments on some reference list

**Rfererence number 4**

“4. World Health Organization. Consolidated guidelines on HIV prevention, diagnosis, treatment and care for key populations: 2016 update. 2016.” in the previous version is amended as:

“7. World Health Organization. Consolidated guidelines on HIV prevention, diagnosis, treatment and care for key populations: World Health Organization; 2016. Available from: <https://www.who.int/publications/i/item/9789241511124>.”

**Rfererence number 9**

“9.	World Health Organization. Guidelines: updated recommendations on HIV prevention, infant diagnosis, antiretroviral initiation and monitoring. World Health Organization, 2021 9240022236.” in the previous version is amended as:

“10.	World Health Organization. Updated recommendations on HIV prevention, infant diagnosis, antiretroviral initiation and monitoring: World Health Organization; 2021. Available from: <https://www.who.int/publications/i/item/9789240022232>.”

**Rfererence number 11**

“11.	Federal Ministry of Health Ethiopia. National Consolidated Guidelines for Comprehensive Hiv Prevention, Care and Treatment. Ethiopia, Addis Ababa: Federal Ministry of Health, Ethiopia; 2018.” in the previous version is amended as:

“12.	Federal Ministry of Health Ethiopia. National Consolidated Guidelines for Comprehensive Hiv Prevention, Care and Treatment. Ethiopia, Addis Ababa: Federal Ministry of Health, Ethiopia; 2018. Available from: <https://www.afro.who.int/publications/national-consolidated-guidelines-comprehensive-hiv-prevention-care-and-treatment>.”

**Rfererence number 12**

“12.	World Health Organization. Consolidated guidelines on the use of antiretroviral drugs for treating and preventing HIV infection: recommendations for a public health approach. World Health Organization,, 2016 9241549688.” in the previous version is amended as:

“4.	World Health Organization. Consolidated guidelines on the use of antiretroviral drugs for treating and preventing HIV infection: recommendations for a public health approach: World Health Organization; 2016. Available from: <https://www.who.int/publications/i/item/9789241549684>.”

**Reviewer #1:**

Most of the previous comments are well addressed, but from the introduction section is not answered well, unless revised or amend it I regret to procced to next step (accept for publication). So please narrate the introduction section at most to 2 to 4 pages.

**Response:**

Thank you for your feedback on my paper. We appreciate your suggestion regarding the length of the introduction section.

Considering the feedbacks given by you we try to summaries and resynthesis the introduction part within the given page limit. We appreciate your time and effort in reviewing my paper and welcome any further feedback or suggestions you may have.
